# Supplementary material for: Label-Free Human Disease Characterization through Circulating Cell-Free DNA Analysis Using Raman Spectroscopy
Source: Int J Mol Sci. 2023 Aug 3;24(15):12384. doi: 10.3390/ijms241512384 (PMC10418917; doi:10.3390/ijms241512384)
Supplement: Supplementary file 1 [file ijms-24-12384-s001.zip › ijms-2517307-supplementary.pdf]

Label-Free Human Disease Characterization through Circulating Cell-Free DNA Analysis Using Raman Spectroscopy

Supplementary Files

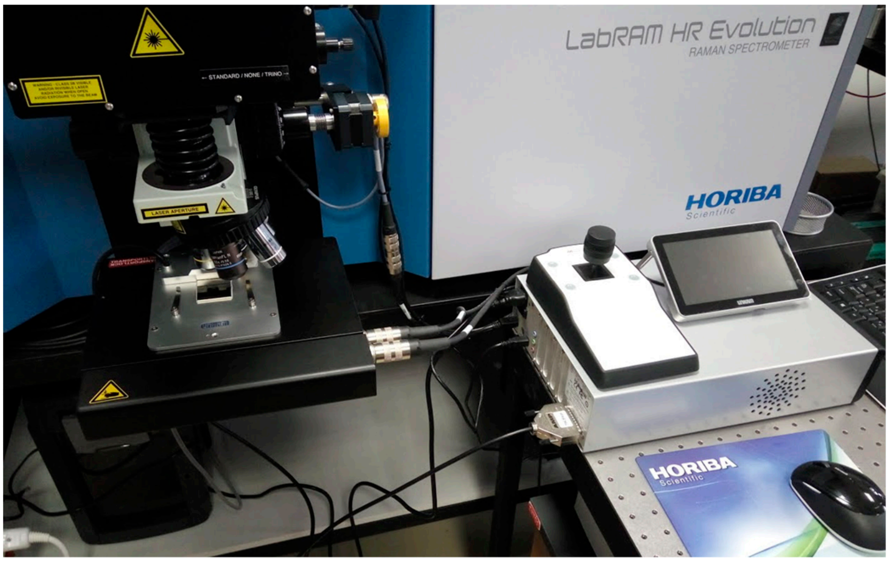

Supplementary Figure S1. The experimental setup used. The Raman microscope, the temperature control stage and the objective lens are depicted.

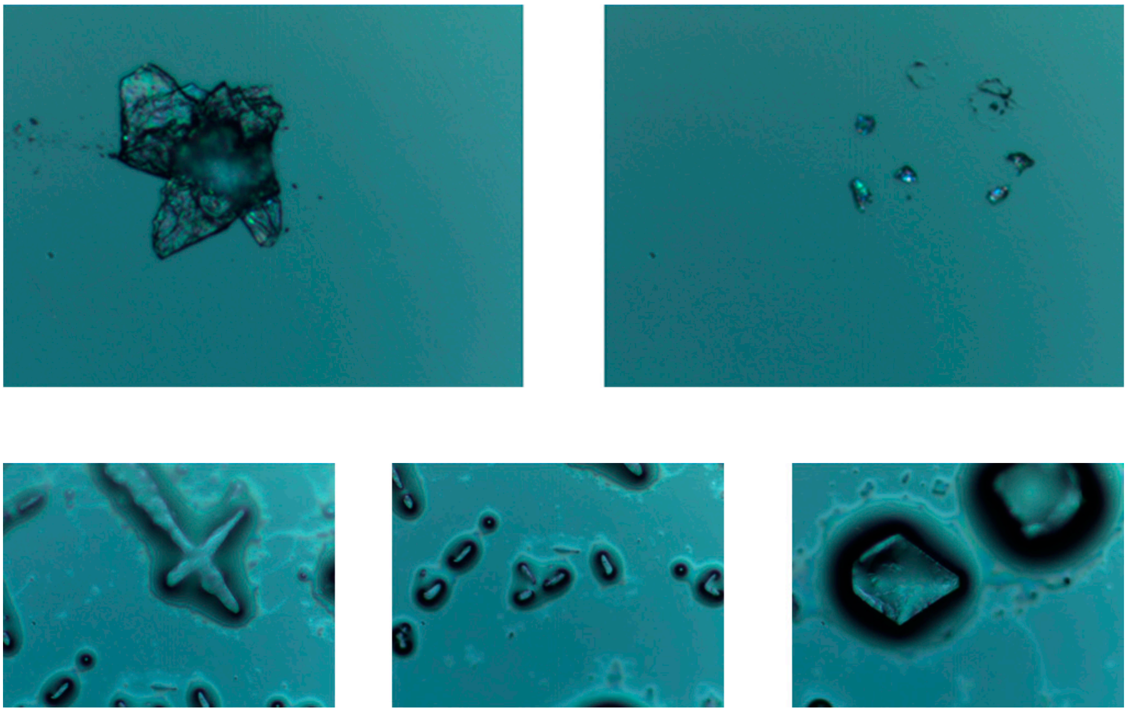

Supplementary Figure S2. Dried DNA cluster images on CaF2 substrate. Microscope magnification was 50X.

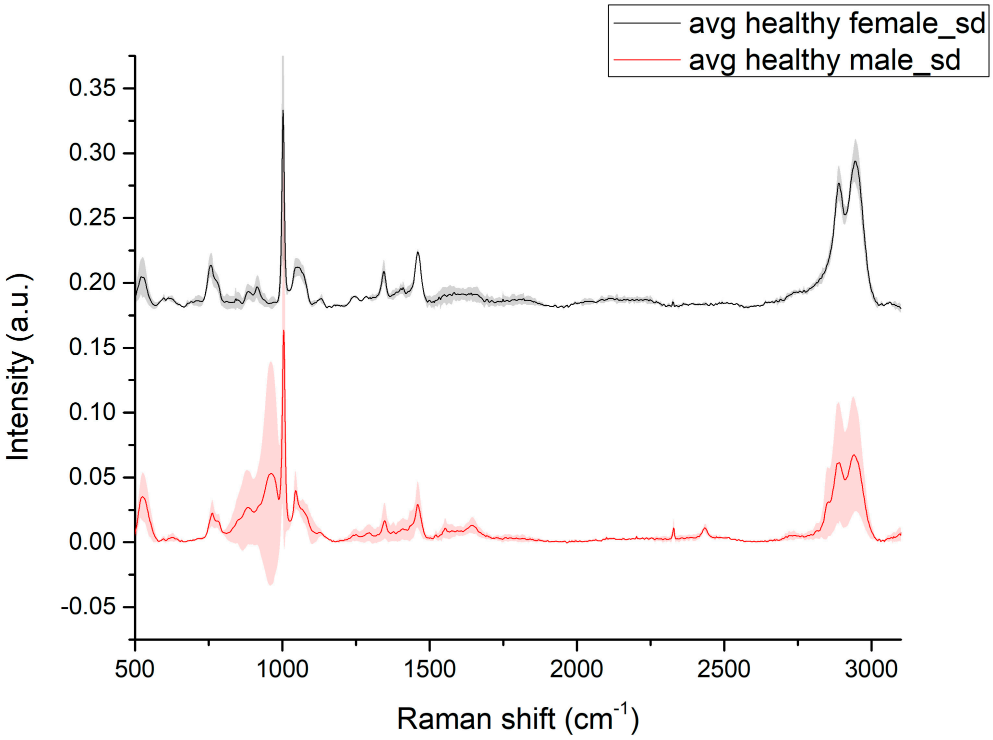

Supplementary Figure S3. Stack-line of the average Raman spectra together with the standard deviation (SD) is presented in each curve. The male subjects are presented in red, while female subjects are in black color.

Supplementary Table S1. Table of clinical and demographic information of healthy individuals and patients included in the study.

Patient group	BMI	Sex	ccfDNA concentration in plasma (ng/ml)	ccfDNA concentration after extraction (ng/µl)	Treatment
Healthy n=6<br>Average Age 62 yo	n/a	F	n/a	0.34	n/a
	n/a	F	n/a	0.274	n/a
	n/a	F	n/a	4.44	n/a
	29.32	M	680	0.144	n/a
	26.12	M	580	0.23	n/a
	21.34	M	920	0.434	n/a
Metastatic Breast cancer patients<br>(=11)<br>Average age 61 yo	n/a	F	616	0.202	Taxoprol,carbo,<br>Retacrit,arimidex,
	n/a	F	780	0.204	Abraxane, avastin, zometta
	n/a	F	475	n/a	n/a
	n/a	F	569	n/a	n/a
	n/a	F	3000	n/a	n/a
	n/a	F	493	n/a	n/a
	n/a	F	643	n/a	n/a
	23,01	F<br>1st measurement	552	0.136	n/a

		2nd measurement	623	0.192	n/a
		F 1st measurement	649	0.21	n/a
	32.47	2nd measurement	579	0.18	n/a
		F 1st measurement	586	0.16	n/a
	39.64	2nd measurement	431	0.596	n/a
		F 1st measurement	527	0.208	n/a
	29.76	2nd measurement	596	0.132	n/a
		F	668	0.276	n/a
Breast cancer patients taking adjuvant therapy (=9) Average age 57 yo	n/a	F	1110	10	n/a
	n/a	F	470	n/a	n/a
	n/a	F	521	n/a	n/a
	n/a	F	569	n/a	n/a
	n/a	F	242	n/a	n/a
	n/a	F	323	n/a	n/a
	n/a	F	469	0.164	n/a

		1st measurement			
	19	2nd measurement	379	0.312	n/a
		F<br>1st measurement	456	0.174	n/a
		2nd measurement	479	0.156	n/a
		F	765	n/a	n/a
Breast cancer patients taking neoadjuvant therapy (=5)<br>Average age 44 yo	n/a	F	253	n/a	n/a
	n/a	F	932	n/a	n/a
	n/a	F	803	n/a	n/a
	n/a	F	673	n/a	n/a
	n/a	M	n/a	n/a	n/a
Prediabetic patients (=3)<br>Average age 40 yo	36.46	M	n/a	n/a	n/a
	28.06	M	n/a	n/a	n/a
	28.03	M	900	0.448	Glucophage
Diabetic patients (n=3)<br>Average age 60 yo	30.61	M	740	1.44	Glucophage
	35.7	M	670	0.156	Glucophage
	26.44				
